# Supplementary material for: The interactome of intact mitochondria by cross-linking mass spectrometry provides evidence for coexisting respiratory supercomplexes
Source: Mol Cell Proteomics. 2017 Dec 8;17(2):216–32. doi: 10.1074/mcp.RA117.000470 (PMC5795388; doi:10.1074/mcp.RA117.000470)
Supplement: Supplemental Data [file supp_RA117.000470_133922_0_supp_23978_fzffwf.zip › spectra_annotation/mito_DR_spectra_annotation/284-1-1-1-6-1.pdf]

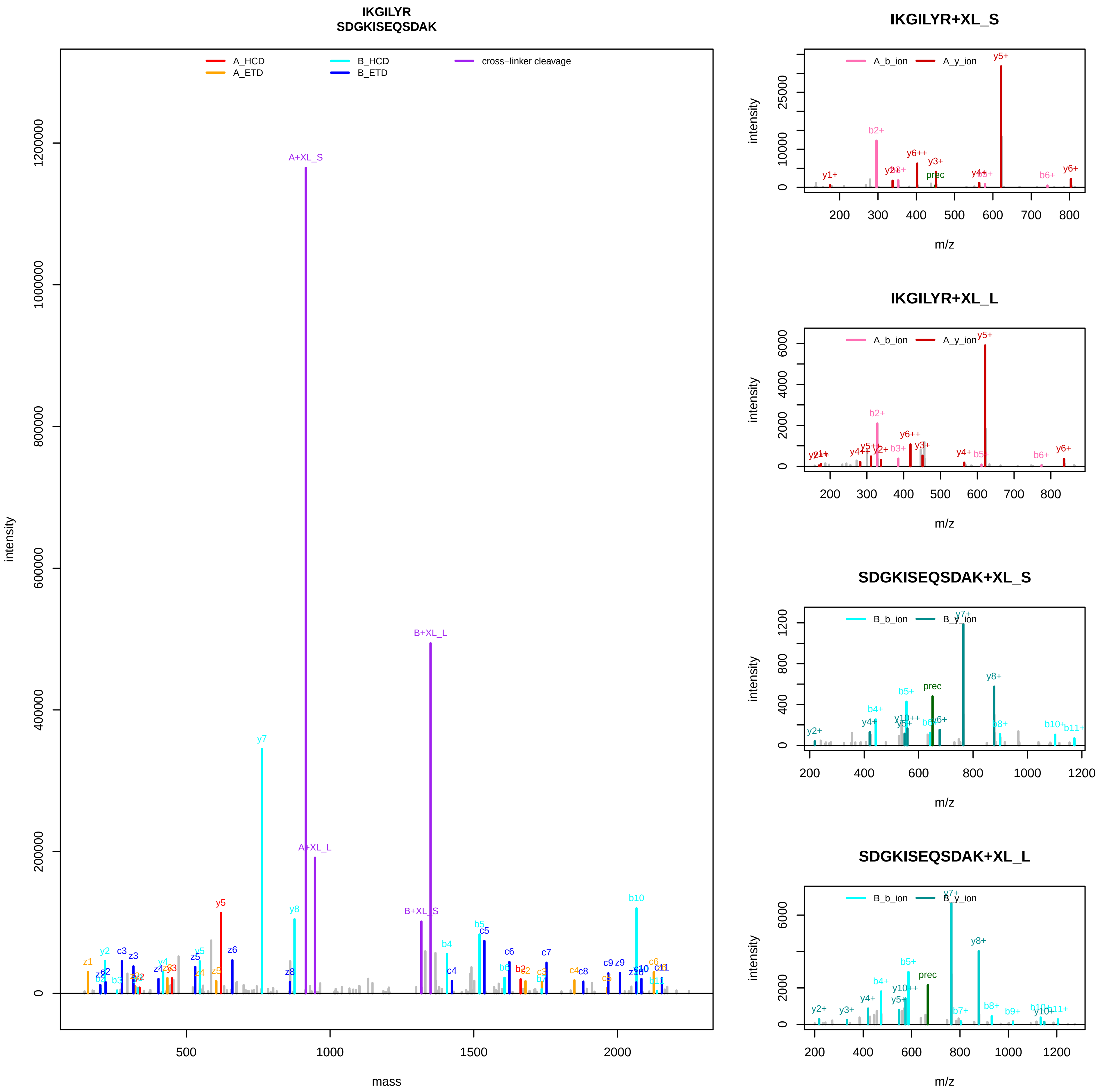
IKGILYR
SDGKISEQSDAK
A_HCD
A_ETD
B_HCD
B_ETD
cross−linker cleavage
A+XL_S
A+XL_L
B+XL_S
B+XL_L
y7
y8
y5
b10
b5
c5
b4
c4
c6
c7
c8
c9
z9
z10
c10
c11
b11
z1
z2
z3
z4
z5
z6
z8
y2
y3
y4
y5
b2
b3
b6
b7
c2
c3
intensity
mass
0
200000
400000
600000
800000
1000000
1200000
500
1000
1500
2000
IKGILYR+XL_S
A_b_ion
A_y_ion
y5+
b2+
y6++
y3+
prec
y1+
y4+
b5+
b6+
y6+
0
10000
25000
200
300
400
500
600
700
800
m/z
IKGILYR+XL_L
A_b_ion
A_y_ion
y5+
b2+
y6++
y3+
b3+
y4+
b5+
b6+
y6+
0
2000
4000
6000
SDGKISEQSDAK+XL_S
B_b_ion
B_y_ion
y7+
y8+
prec
b5+
b4+
y4+
y10++
y6+
b10+
b11+
y2+
0
400
800
1200
200
400
600
800
1000
1200
SDGKISEQSDAK+XL_L
B_b_ion
B_y_ion
y7+
y8+
prec
b5+
b4+
y4+
y10++
y5+
y3+
y2+
b7+
b8+
b9+
b10+
y10+
b11+
0
2000
6000
